# Discovery of Two Potential and Novel Toll-like Receptor 7 Antagonists via Molecular Docking, Molecular Dynamics Simulation and In Vitro Biological Evaluation

**DOI:** 10.3390/molecules31213903

**Published:** 2026-10-29

**Authors:** Chang Liu, Zhe Qin, Lixia Bai, Xiao Xu, Wenbo Ge, Zhun Li, Jianyong Li

**Affiliations:** Key Laboratory of New Animal Drug of Gansu Province, Key Laboratory of Veterinary Pharmaceutical Development of Ministry of Agriculture and Rural Affairs, Lanzhou Institute of Husbandry and Pharmaceutical Sciences of CAAS, Lanzhou 730050, China

**Keywords:** virtual screening, molecular docking, molecular dynamics simulation, biological evaluation, novel TLR7 antagonists

## Abstract

Objective: Toll-like receptor 7 (TLR7) is an important pattern recognition receptor. It mainly participates in the recognition of single-stranded RNA and is closely related to various disease processes. In humans, the enormous potential of TLR7 antagonists in reducing inflammation and alleviating autoimmune damage has been identified. Similar effects have also been observed in livestock and poultry. Therefore, using porcine TLR7 as the target, we combined virtual screening with a heterologous reporter gene assay to identify potential ligands, aiming to provide a research basis for the development of immune modulators. Methods: The structure of porcine TLR7 was predicted through homology modeling, and molecular docking was performed for virtual screening of potential ligands. ADMET and toxicity properties of small molecules were predicted. Subsequently, a mouse TLR7 reporter cell was employed for in vitro detection of TLR7 activity and cytotoxicity. The interaction modes and molecular dynamics simulations between hit compounds and receptors were further analyzed. Results: The porcine TLR7 structure was predicted by homology modeling. According to the scoring ranking of molecular docking and of ADMET and toxicity prediction, fifty compounds with satisfactory pharmacokinetic properties and safety were screened and their inhibitory effects on R848-induced TLR7 activation were verified. Among them, two hit compounds with novel structures, HY02-35 and HY02-46, exhibited significant mouse TLR7 antagonistic activity. HY02-35, with a parent nucleus structure of 2-indolone, exhibited an inhibitory concentration range between 10.25 and 38.23 µM, with a median estimate of 14.73 µM. The core structure of HY02-46 is 1,4-dihydropyrano [2,3-c] pyrazole, and the IC_50_ falls between 21.92 and 36.00 µM, with a median estimate of 26.61 µM. The above-mentioned hit compounds had strong interactions with porcine TLR7, formed stable complexes with the receptor, and can significantly reduce the mRNA levels of IFN-β and TNF-α. Conclusions: In short, this study obtained two hit compounds with novel structures and moderate inhibitory activity in vitro. These hits provide a starting point for subsequent structural optimization and in vivo pharmacological studies in porcine subjects.

## 1. Introduction

Toll-like receptors (TLRs) are a series of pattern recognition receptors (PRRs) involved in the recognition of pathogen-associated molecular patterns (PAMPs) or damage-associated molecular patterns (DAMPs), connecting innate and adaptive immune responses [1,2,3]. Among them, TLR7 is located in the endosome membrane and participates in the recognition of single-stranded RNA (ssRNA) [4]. Similar to other TLR members, the domain architecture of TLR7 consists of three parts: the extracellular domain, transmembrane domain, and Toll/Interleukin-1 receptor (TIR) domain [5,6].

TLR7 activation stimulates the secretion of proinflammatory cytokines [7,8,9,10] and initiates a MyD88-dependent signaling cascade that activates signaling pathways such as NF-κB and mitogen-activated protein kinase (MAPK), as well as phosphoinositol 3-kinase/serine/threonine protein kinase (PI3K/AKT) [11,12]. However, abnormal activation of TLR7 is associated with various inflammatory and autoimmune diseases. TLR7 antagonists can reduce the secretion of proinflammatory cytokines and alleviate symptoms of related diseases by preventing TLR7 activation. On the one hand, TLR7 antagonists are conducive to the enhancement of anti-tumor effects via their regulation of immune responses and their inhibition of tumor-induced cachexia [13,14]. On the other hand, TLR7 antagonists are also effective for autoimmune diseases such as systemic lupus erythematosus, rheumatoid arthritis, inflammatory bowel disease, colitis, etc. [15,16]. In addition, TLR7 ligands have shown great potential in separating X/Y sperm and controlling sex in livestock and poultry, and they have enormous value for animal husbandry [17,18]. Since the emergence of Imiquimod (R837) [11,19], researchers have designed and synthesized various compounds with a TLR7 activation effect, including purine [20] and pyrimidine [21] derivatives, imidazo[4,5-c]quinoline [22], pyrido[2,3-d]pyrimidine [23] and methotrexate [24,25].

However, research on TLR7 antagonists in the field of livestock and poultry disease prevention is still insufficient. Furthermore, the response of TLRs to ligands varies among different species [26]. The crystal structure of porcine TLR7 has not yet been elucidated, and the development of animal-specific TLR7 antagonists and their application in livestock and poultry disease prevention are in need of further exploration. Therefore, this study targeted porcine TLR7 and used a structure-based virtual screening method to screen potential antagonists of porcine TLR7. Based on ADMET (absorption, distribution, biotransformation, excretion, and toxicity) prediction, TLR7 reporter cell assay and molecular dynamic (MD) simulation, two hit compounds with a novel structure were identified. The results will provide reference for the development of TLR7 ligands as livestock and poultry immune regulators.

## 2. Results

### 2.1. Template Identification and Sequence Similarity

**Figure 1:**
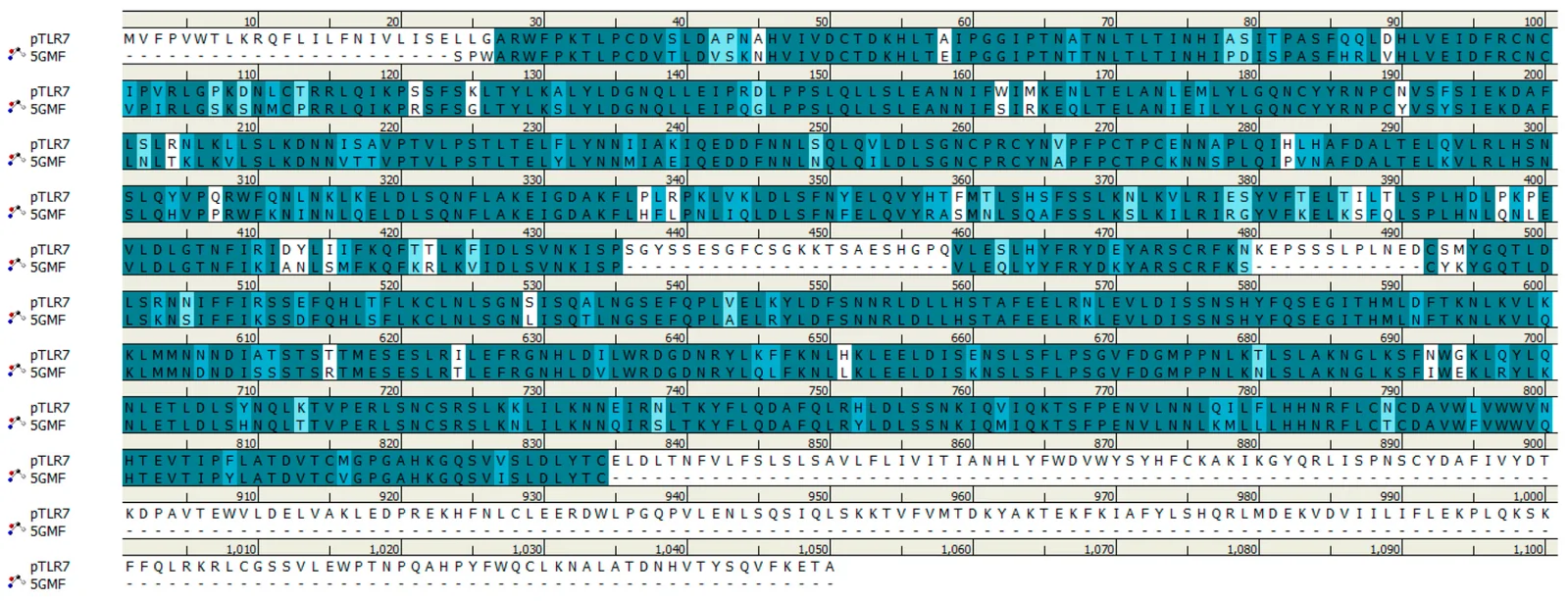
Sequence alignment between porcine TLR7 and template (PDB ID: 5GMF).

Generally, when the identity between target sequence and template sequence in homology modeling is higher than 30%, the result of protein homology modeling has higher accuracy [27,28,29]. According to the result of BLAST research, monkey TLR7 (PDB ID: 5GMF) was selected as the modeling template. The crystal structure of 5GMF was determined by X-ray diffraction at a resolution of 2.5 Å, in complex with guanosine and polyU, representing the agonist-bound conformation of TLR7. It was shown that protein 5GMF contains 817 amino acids, with a percent identity of 81.08% and a query cover of 77% with porcine TLR7. The alignment result between target protein and 5GMF was with sequence identity of 76.0% and sequence similarity of 85.9% (Figure 1).

### 2.2. Homologous Modeling and Model Reliability Evaluation

#### 2.2.1. GMQE and QMEAN

GMQE and QMEAN are validation parameters used to evaluate the accuracy and stereochemical properties of predictive models.

**Figure 2:**
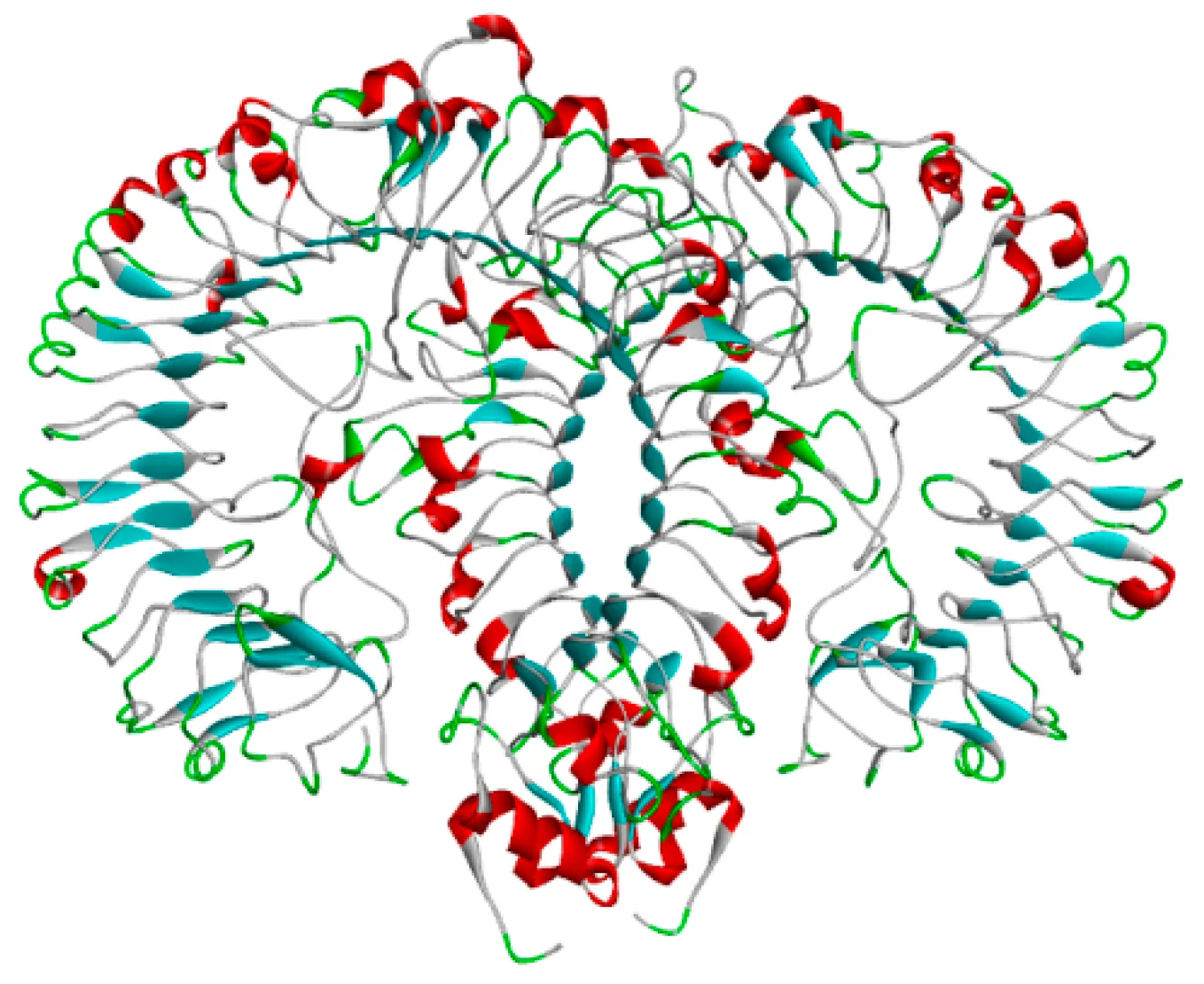
Predicted structure of porcine TLR7. The structure was colored by secondary type, with helices in red, beta sheets in cyan, turns in green, and coils in white.

GMQE represents the expected accuracy of the model and the coverage range of the target protein. The score of GMQE is represented as a value between 0 and 1. The closer the score is to 1, the higher the reliability of the constructed model [30,31]. QMEAN is a composite scoring function used to evaluate the quality of protein structure models based on their geometric and physical features. The score of QMEAN ranges from −4 to 0. The closer the score is to 0, the more it conforms to the rules of stereochemistry, which indicates good consistency between the predicted model and experimental structures. A score close to or below −4.0 indicates inferior quality [31]. The modeling structure obtained from SWISS-MODEL was one with a GMQE score of 0.59 and the QMEAN score of−2.49 (Figure 2).

#### 2.2.2. Ramachandran Plot

**Figure 3:**
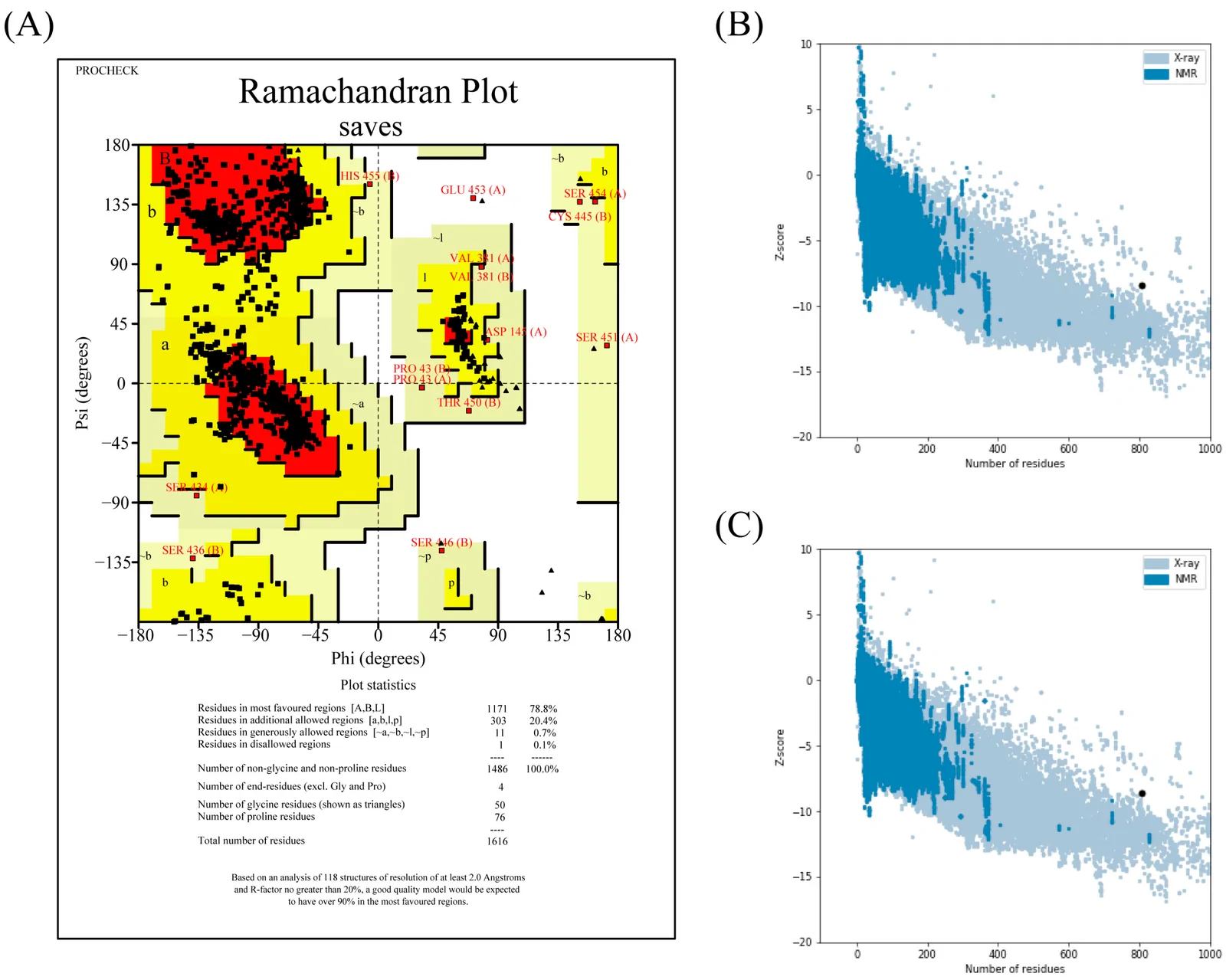
Validation for homology modeling. (**A**) Ramachandran plot of the predicted structure of porcine TLR7. The characters a, B, b, ~b, p, ~p, l and ~l represent allowed alpha, core beta, allowed beta, generous beta, allowed epsilon, generous epsilon, allowed left-handed alpha and generous left-handed alpha, respectively. And the colors red, yellow, cream and white mark most favoured region, additional allowed region, generously allowed region and disallowed region, respectively. (**B**) and (**C**) show the Pro-SA plots of chain A and chain B, respectively.

A Ramachandran plot represents the degree of rotation (psi) of the bond between the alpha carbon atom and the carbonyl carbon atom, as well as the degree of rotation (phi) of the bond between the alpha carbon atom and the nitrogen atom in the protein 3D structure. It was used to indicate allowed and disallowed conformations of amino acid residues in protein, evaluate the rationality of the conformation of two adjacent peptide unit skeletons, and determine whether the conformation of each amino acid in the structure is correct [31]. A Ramachandran plot of the predicted structure is shown in Figure 3A. A total of 78.8% of amino acids were distributed in the most favored regions, 20.4% in additional allowed regions, 0.7% in generally allowed regions, and 0.1% in disallowed regions. Nearly all of the residues with unfavorable conformations were not part of the orthosteric site.

#### 2.2.3. Verify Protein (Profiles-3D)

Verify Protein (Profiles-3D) is a model evaluation program based on the “threading” method. This method detects the matching relationship between the constructed model and its own amino acid sequence by calculating a self-compatibility score [32]. The closer the Verify score of the model is to the Verify expected high score, the better the quality. The Verify score was 699.6, which was higher than the Verify expected low score (335.393) and close to the Verify expected high score of 745.318. These results indicate that the predicted structure has high quality. Therefore, the structure was considered reliable and was used for subsequent research.

#### 2.2.4. Pro-SA

Based on statistical analysis of available protein structures, ProSA is a diagnostic tool that is commonly used to validate potential errors in 3D models of protein structures [30]. The evaluation result reflects the interaction energy between amino acid residues in a three-dimensional structure of the target protein, and the calculated value is usually represented as the Z-score. When the Z-score values of the target protein are distributed within the range of Z-score values for these known proteins, it proves that the energy of the target protein structure is reasonable.

The Z-score of chain A and chain B was −8.6 and −8.39, respectively, which falls within a reasonable range. The Pro-SA diagrams are depicted in Figure 3B,C.

### 2.3. Molecular Docking

Molecular docking is one of the structure-based virtual screening methods, and it plays a critical role in the preliminary stage of drug discovery [33,34]. It mainly achieves the purpose of screening by predicting the binding mode of extensive ligands within a given binding pocket. 5GMF was re-docked to validate the molecular docking protocol, with an RMSD of less than 2.0 Å (0.60 Å). So as to find potential ligands for porcine TLR7, small molecules with diverse and unique structure according to the Specs HTS Compounds Library were docked. The HTVS, SP, and XP modes of Glide dock were sequentially performed. Potential ligands with top 200 rankings were filtered out according to their docking scores.

### 2.4. ADMET Analysis and Drug-likeness Properties Prediction

Previous studies confirmed that conducting drug-likeness evaluation in the early stages can save time, reduce costs, and improve efficiency in the drug discovery process [35]. Lipinski’s rule of five states that small-molecule drugs should meet the following criteria: molecular weight (MW) ≤ 500 Da, number of hydrogen bond donors (HBDs) ≤ 5, number of hydrogen bond acceptors (HBAs) ≤ 10, and partition coefficient (LogP) < 5 [36]. According to Veber’s rule, if the topological polar surface area (TPSA) is no less than 140 Å and the number of rotatable bonds ≤ 10, the compound is considered to have good oral bioavailability [37]. Among the 200 top ranking compounds, five compounds violated Lipinski’s rule or Veber’s rule.

**Figure 4:**
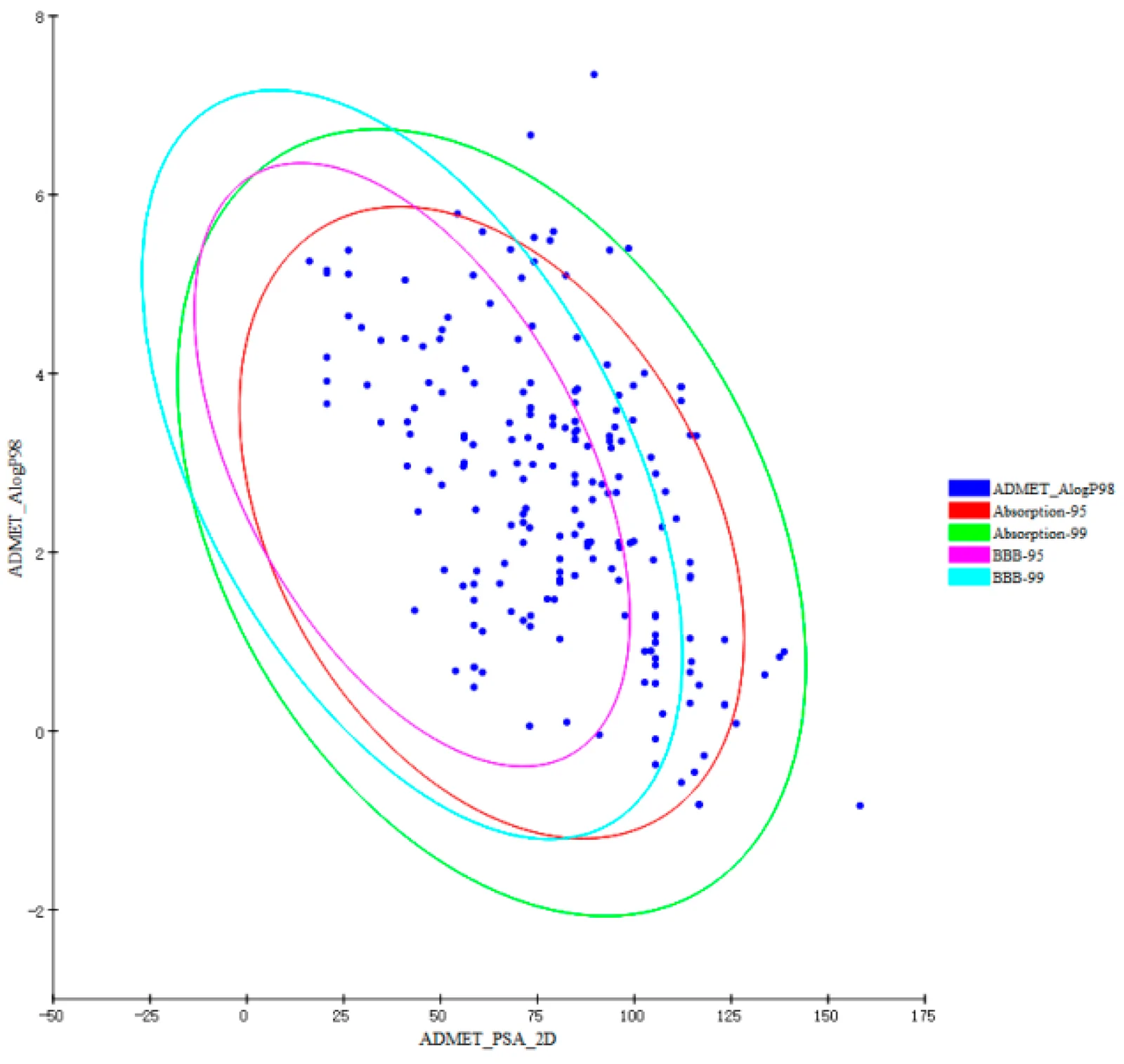
AlogP98 versus two-dimensional polar surface area (PSA_2D) point plot of top 200 compounds. The X axis represents ADMET_PSA_2D, with the unit being Å2, while the Y axis represents ADMET_AlogP98, without any unit.

Additionally, unsatisfactory pharmacokinetic properties and toxicity are the main reasons for the failure of candidate drug development. Therefore, in the early stages of drug development, it is necessary to select and optimize lead compounds based on ADMET properties [36,38]. Therefore, ADMET prediction was subsequently performed in Discovery Studio software (Figure 4). The toxicity of the compound and rat oral LD_50_ (median lethal dose) were also predicted. Taking into account factors such as docking score, ADMET properties, and safety, together with availability, 50 compounds were purchased and used for subsequent validation. The predicted physicochemical and ADMET properties of the above compounds are shown in Table A1 and Table A2.

### 2.5. HY02-35 and HY02-46 Exhibited Potential TLR7 Inhibitory Activity

Due to the unavailability of a porcine TLR7 reporter cell, a mouse TLR7 reporter cell was used for preliminary validation of TLR7 activity. Previous studies have proven that resiquimod (R848) is a dual TLR7/8 agonist in human and mouse subjects. Consequently, R848 was adopted as positive control and Z-factor was calculated to evaluate the quality of this method. Z-factor is a parameter that evaluates whether the method meets further research requirements by comparing positive and negative controls. Its calculation formula is as follows:

(1) $$Z-\mathrm{factor}=1-\frac{3\times (\sigma_{p}+\sigma_{n})}{\left(\mu_{p}-\mu_{n}\right)}$$

where σ_p_ and σ_n_ represent the standard deviations of positive and negative controls, respectively; μ_p_ and μ_n_ represent the average values of positive and negative controls, respectively.

**Figure 5:**
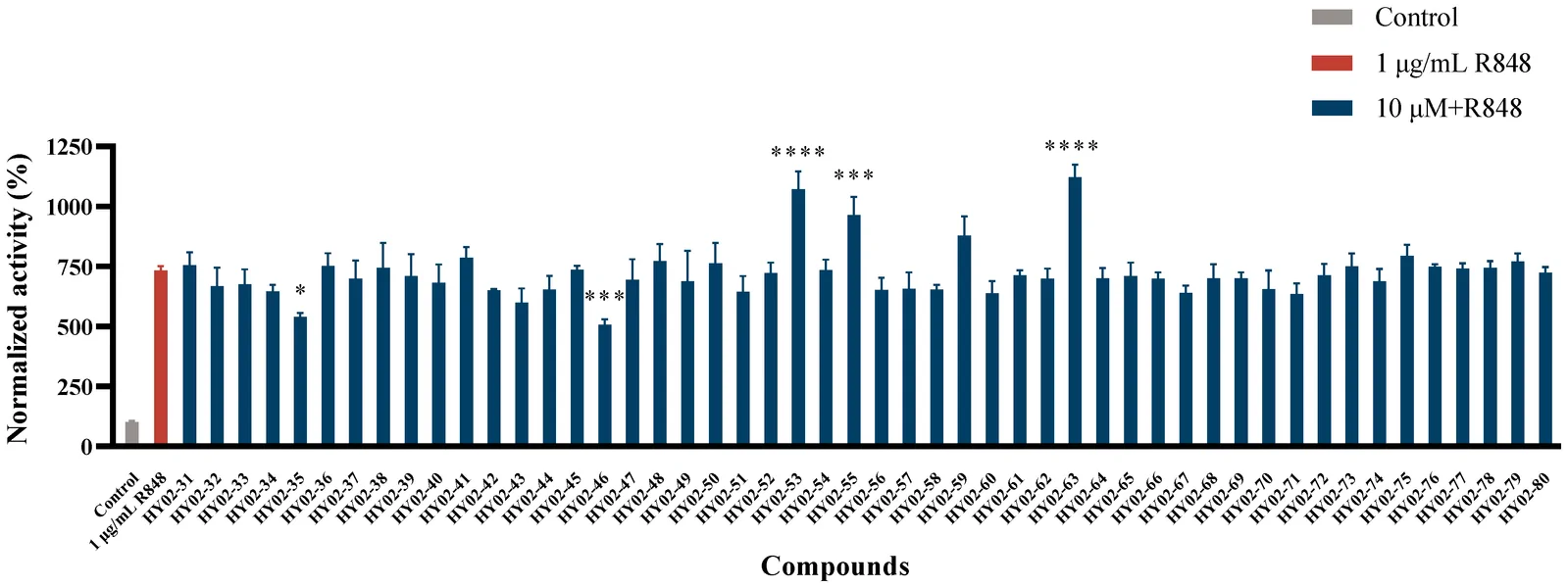
Inhibition effect of fifty selected compounds on R848-induced TLR7 activation at concentration of 10 μmol/L. The unstimulated group served as the negative control, while the group treated with 1 μg/mL R848 served as the positive control. Luminescence was normalized to untreated cells, and results are presented as mean ± SD (*n* = 3). Statistical analysis was performed by one-way ANOVA with Dunnett’s post hoc test. * represents *p* < 0.05, *** represents *p* < 0.001, and **** represents *p* < 0.0001.

In the first round of validation, the cells were incubated with R848 and compounds at a concentration of 10 μmol/L to evaluate the regulatory effect [39]. The TLR7 regulatory effect of 50 selected compounds is illustrated in Figure 5, with a Z-factor of 0.79. Among the above compounds, two compounds (HY02-35 and HY02-46) significantly inhibited TLR7 activation induced by R848, while three compounds (HY02-53, HY02-55 and HY02-63) showed an agonist effect. Subsequently, molecules with significant inhibitory activity and stable repeatability were picked out and further validated. According to IUPAC nomenclature, the compound HY02-35 was named (Z)-4-((5-nitro-2-oxoindolin-3-ylidene)amino)benzonitrile and HY02-46 was named (S)-6-amino-3-(tert-butyl)-4-phenyl-1,4-dihydropyrano [2,3-c]pyrazole-5-carbonitrile.

The mouse TLR7 reporter cell was treated with a series of compounds at different concentrations. The cells were incubated for 24 h prior to the addition of CCK-8 reagent and/or luciferase assay reagent. Absorbance and/or luminescence were detected to investigate the antagonistic effect, with IC_50_ (half-maximal inhibitory concentration) curves being generated. HY02-35 and HY02-46 inhibited TLR7 activation in a dose-dependent manner. The median effective concentration of HY02-35 is approximately 14.73 µM, but the 95% confidence interval is broad (ranging from 10.25 to 38.23 µM). The inhibitory concentration range of HY02-46 is 21.92 to 36.00 µM, with a median estimate of 26.61 µM. It is speculated that the wide confidence interval (CI) is mainly attributed to the lack of a clear plateau period within the tested concentration range. Due to the cytotoxicity of compounds, their inhibitory activity on mouse TLR7 reporter cells at higher concentrations cannot be accurately reflected.

Cell viability was measured at the same time following treatment with various drug concentrations. Both compounds exhibit a certain degree of cytotoxicity at higher concentrations. Cell viability rates of 45.55% and 73.22% were observed following treatment with 25 μmol/L and 50 μmol/L of HY02-35, respectively. As for HY02-46, 100 μmol/L treatment resulted in 54.36% viability and 50 μmol/L treatment led to 82.19% viability.

**Figure 6:**
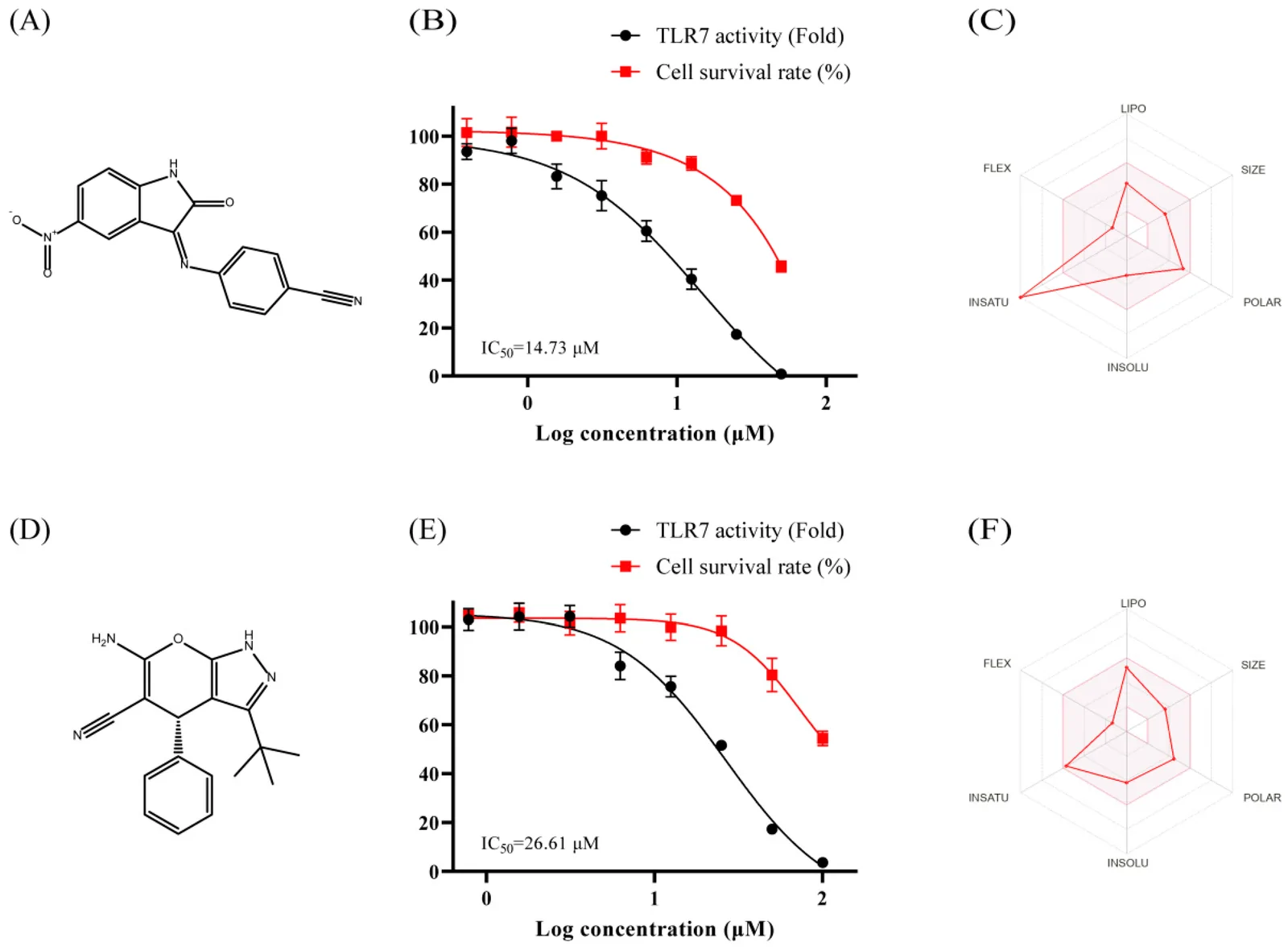
Antagonistic activity of compounds on mouse TLR7. The structures, IC_50_ curves and bioactivity radar charts of compounds HY02-35 (**A**–**C**) and HY02-46 (**D**–**F**) are presented in sequence (*p* < 0.0001). Luminescence was normalized to untreated cells, and results are presented as mean ± SD (*n* ≥ 3).

Therefore, two preliminary validated active compounds mentioned above have the potential to serve as TLR7 antagonists. However, both compounds showed a certain degree of cytotoxicity in vitro. In future research, it is necessary to modify their structure to reduce toxicity, while enhancing activity, so that they can serve as more effective TLR7 modulators. The chemical structures, corresponding IC_50_ curves and cell viability of HY02-35 and HY02-46 are shown in Figure 6.

Both HY02-35 and HY02-46 exhibit high predicted GI (gastrointestinal) absorption without P-glycoprotein (P-gp) substrate binding (Table 1). The LogP of HY02-35 and HY02-46 was 1.71 and 3.49, respectively, indicating the potentially stronger membrane permeability of the latter [40]. Moderate LogP and TPSA suggest that two hit compounds may have good oral bioavailability [41]. The bioavailability radar charts were generated using the SwissADME platform (Figure 6C,F). Most descriptors of hit compounds fall within the optimal pink area, indicating overall compliance with drug-likeness criteria. However, a high degree of unsaturation was observed in HY02-35, suggesting that the molecule has a high rigidity and may be metabolically unstable.

### 2.6. Interaction Analysis

**Figure 7:**
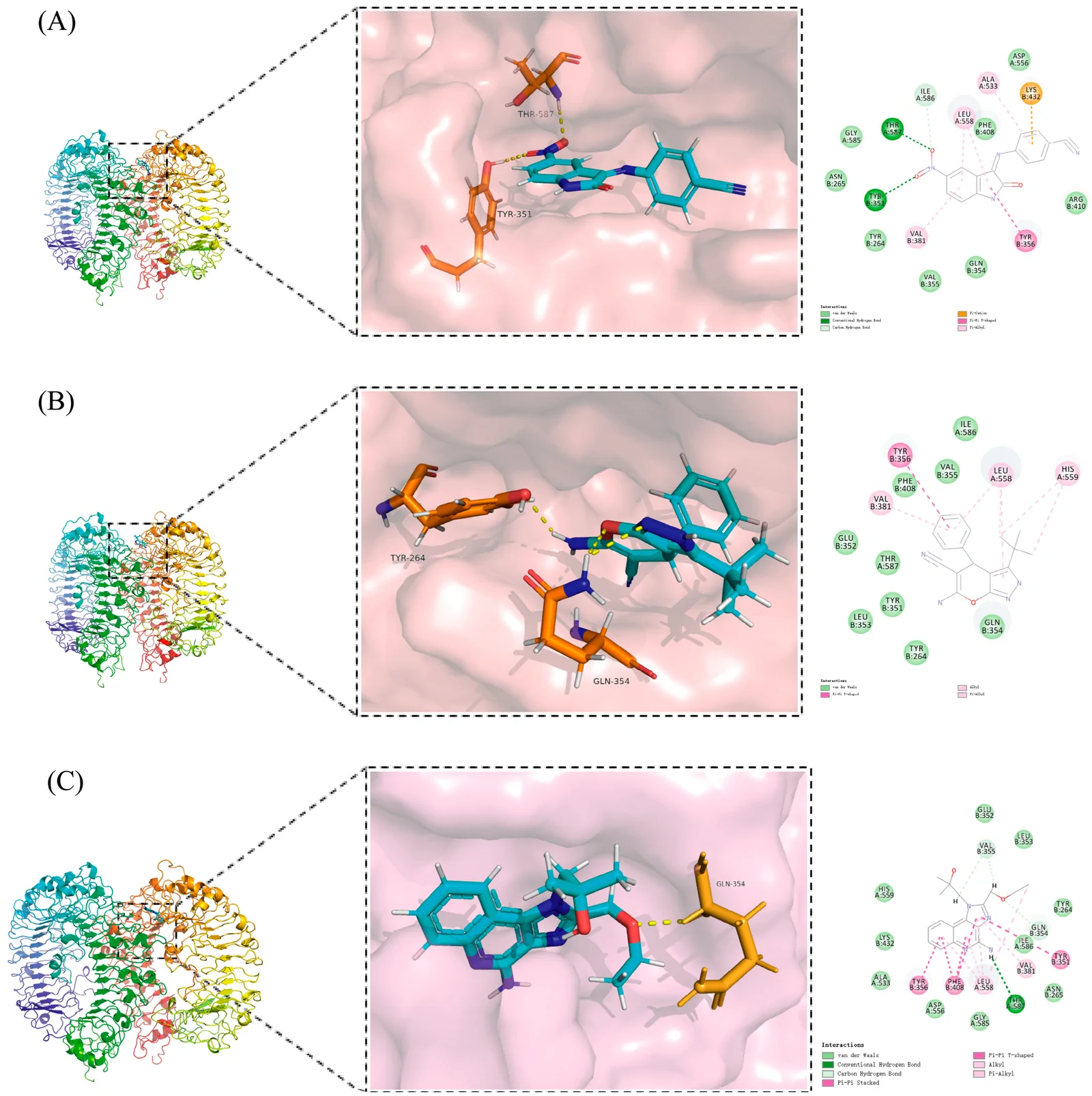
Interaction between porcine TLR7 and two hit compounds and R848. (**A**) Compound HY02-35. (**B**) Compound HY02-46, (**C**) R-848.

The two compounds mentioned above exhibited an antagonist effect. The interaction mode between receptor and ligands was observed (Figure 7) [42,43,44].

The nitro group of compound HY02-35 could interact with residues TYR351*, GLN354*, VAL355* and THR587 of porcine TLR7 through hydrogen bonding, in which the symbol * represents the amino acid located on the B chain of protein dimer. Further, the benzene ring located on the side chain of HY02-35 contacts LYS432* and ASP556 through pi-cation, which is a type of electrostatic interaction. Hydrophobic interactions, such as pi-pi T-shaped and pi-alkyl, were formed between the ligand and residues TYR356*, VAL381*, LEU558 and ALA533. GLY585, ASN265*, TYR264* and other residues participate in the formation of van der Waals forces (Figure 7A).

Hydrogen bonding interaction is also formed between HY02-46 and the receptor. The oxygen atom of pyran and nitrogen atoms of pyrazole group engage in hydrogen bond interactions with VAL355* and THR587. The amino group of HY02-46 forms a hydrogen bond and salt bridge with GLN354* and ASP556, respectively. The phenyl and pyrazole ring forms hydrophobic interactions with residue TYR356*, VAL381*, LEU558 and HIS559, including pi-pi T shaped and pi-alkyl interactions. Residues such as ILE586, VAL355* and PHE408 are related to van der Waals forces (Figure 7B). The above amino acid residues are similar to those involved in the pTLR7-R848 interaction. R848 forms hydrogen bonds with THR587 and GLN354 *, and van der Waals forces form hydrogen bonds with residues such as VAL355*, LYS432* and HIS559. It also exhibits hydrophobic interactions with TYR264*, PHE408* and TYR356* (Figure 7C).

### 2.7. MD Simulation and MM-PBSA Calculation

**Figure 8:**
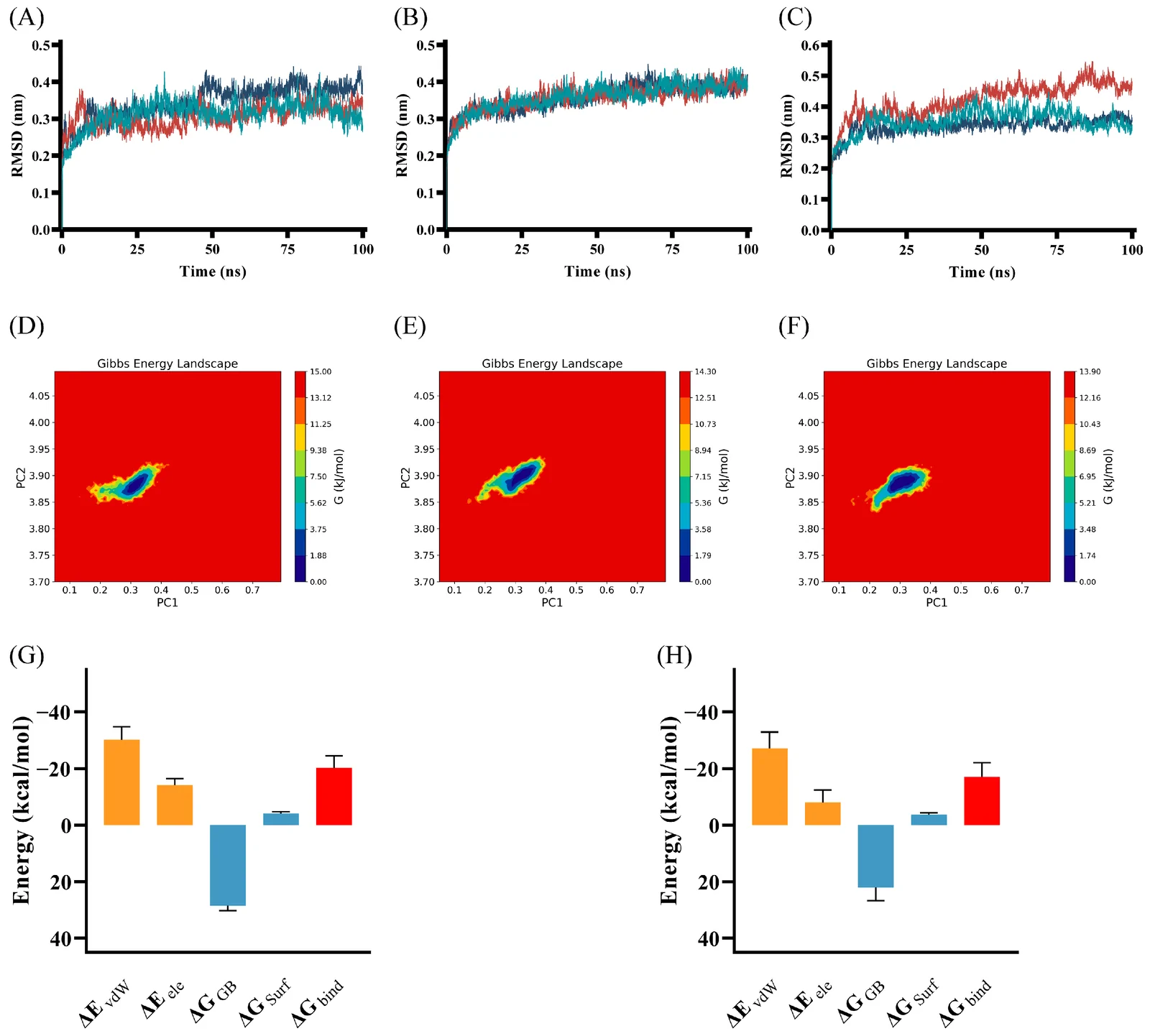
MD simulation analysis of unbounded form of the protein and complexes. (**A**–**C**) are RMSD plots of apo-form and pTLR7-HY02-35 complex, as well as pTLR7-HY02-46 complex with three replicates, respectively. (**D**–**F**) are Gibbs energy landscapes of apo-form and complexes of pTLR7 with HY02-35 and HY02-46, respectively, with one repetition shown. Further, (**G**,**H**) are binding free energy of complexes of pTLR7 with HY02-35 and HY02-46, respectively. Binding free energy was calculated based on three repetition results and is represented as mean ± SD.

**Figure A1:**
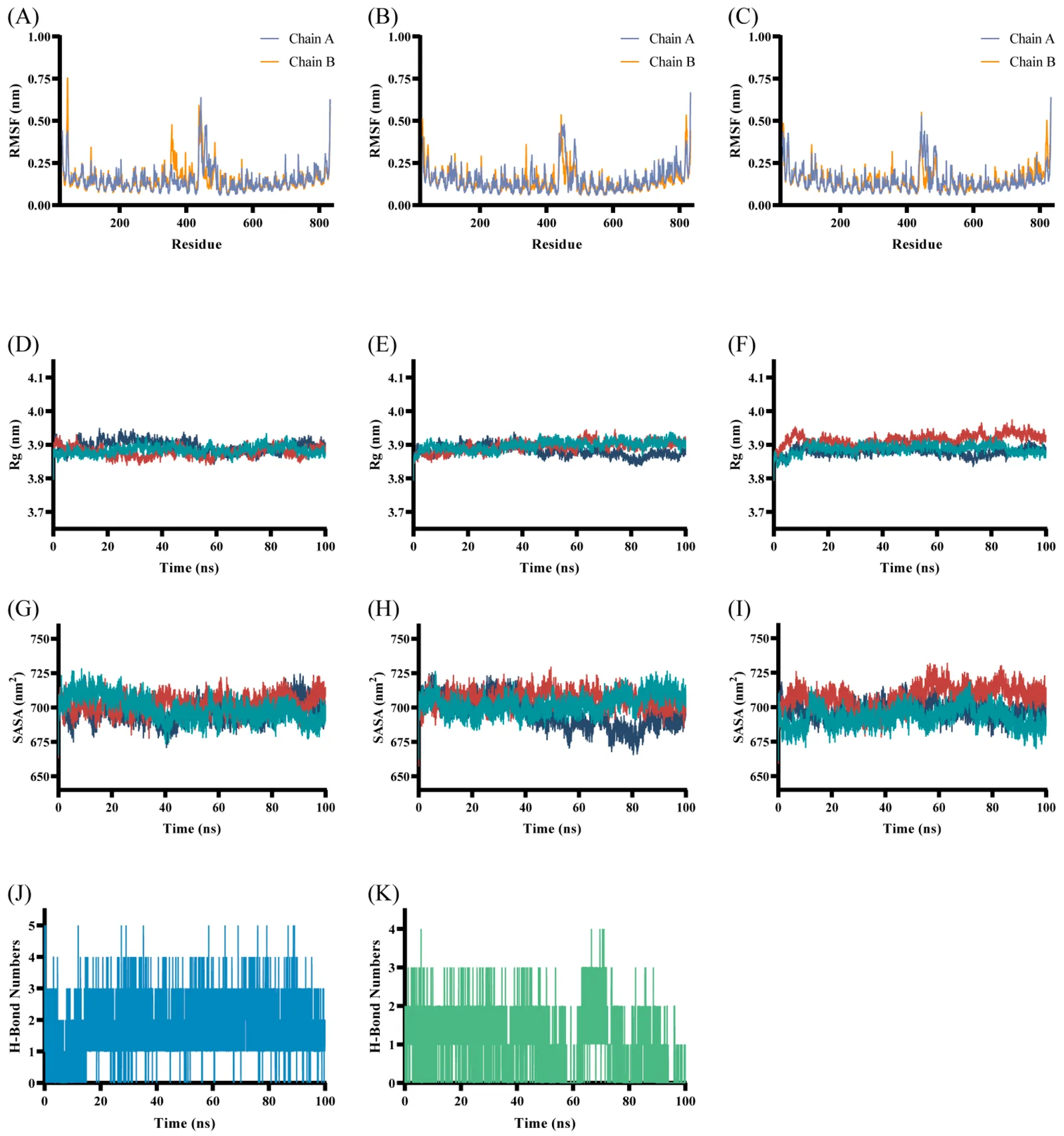
MD simulation analysis of unbounded form of the protein and complexes. (**A**–**C**) are RMSF plots of apo-form, complex of porcine TLR7 with HY02-35, and complex of porcine TLR7 withHY02-46, respectively, with one repeated result depicted. (**D**–**F**) are Rg plots of apo-form, porcine TLR7-HY02-35 complex, and porcine TLR7-HY02-46 complex with three replicates, respectively. (**G**–**I**) are SASA of apo-form, porcine TLR7-HY02-35 complex, and porcine TLR7-HY02-46 complex with three replicates, respectively. (**J**,**K**) are the H-bond number of complexes of porcine TLR7 with HY02-35 and HY02-46, respectively, with one repeated result depicted.

In this study, the MD simulation analysis was conducted for three replications in order to sample adequately and calculate binding free energy accurately. The MD simulations were performed on the apo-form of porcine TLR7 and its complexes with two ligands. Root mean square deviation (RMSD), root mean square fluctuation (RMSFs), radius of gyration (Rg), solvent accessible surface area (SASA) and hydrogen bond number (NHB) were analyzed (Figure 8 and Figure A1). The Gibbs energy landscape was generated, and MM-PBSA was calculated.

#### 2.7.1. RMSD

RSMD is an indicator for assessing the stability of the protein–ligand complex, providing information on the stability of the complex throughout the entire simulation process and reflecting the changes between initial conformation and final conformation [45,46]. As it can be seen, the stability and repeatability of porcine TLR7-HY02-35 complex were better than that of apo-form. The complex formed by HY02-35 and the receptor fluctuated between 0.2 nm and 0.5 nm in the first 20 ns and then gradually stabilized around 0.4 nm (Figure 8B). The deviation in the RMSD curve is lower than that of apo-form, indicating that the binding of HY02-35 makes the complex more stable. Similarly, in the 100 ns simulation, the RMSD of the porcine TLR7-HY02-46 complex fluctuated before gradually stabilizing (Figure 8C). However, a significant structural deviation was observed in certain repetitions of the porcine TLR7-HY02-46 complex, which may be related to its high flexibility. The average and standard deviation of the RMSD of complexes at 60–100 ns are listed in Table 2, with RMSD_pTLR7-apo_, RMSD2_pTLR7-HY02-35_, and RMSD_pTLR7-HY02-46_ being 0.344 ± 0.004 nm, 0.385 ± 0.003 nm, and 0.394 ± 0.006 nm, respectively.

#### 2.7.2. RMSF

RMSF can be used to measure the fluctuation degree of atomic positions in MD simulation, and it is often applied to study the thermal fluctuations of atoms or residues [47,48]. A lower RMSF value indicates less fluctuation of the amino acid residue. On the contrary, a higher RMSF may suggest significant conformational changes in this region. In apo-form, amino acids within the range of 320–380 and 440–500 exhibit obvious fluctuation. Meanwhile, the RMSF decreased upon binding with ligands, indicating that the formed complexes are more stable. The RMSF plot of two complexes showed small fluctuations within 1 nm (Figure A1A–C). The fluctuation of the amino acids of complexes within the range of 440–500 may be associated with the flexibility and interaction with ligands.

#### 2.7.3. Rg

Rg can reflect the shape and size of the system and is an important tool for studying molecular conformation [49]. A larger Rg means that the structure of system is more loose. Meanwhile, a smaller Rg implies a relatively compact system. The Rg diagrams, as well as the average and SD, of apo-form and two complexes are shown in Figure A1D–F and Table 3, respectively. Both Rg values of the complexes remained stable around 3.9 nm (Rg_pTLR7-HY02-35_ = 3.894 ± 0.000 nm, Rg_pTLR7-HY02-46_ = 3.897 ± 0.001 nm), which is comparable to that of apo-form (Rg_apo_ = 3.885 ± 0.001 nm), suggesting that receptor–ligand complexes maintained tightness and stability during the 100 ns simulation process, and ligand binding did not significantly alter the overall compactness of the protein.

#### 2.7.4. SASA

SASA analysis was employed to investigate solvent accessibility. It refers to the surface area of biomolecules that solvent can come into contact with and is used to describe the interaction between molecule and solvent. Proteins or biomolecules with stable structures often have more stable SASAs [50,51]. In the latter part of the trajectory, the SASA of apo-form (SASA_apo_ = 699.679 ± 0.841 nm^2^) and the two complexes (SASA_pTLR7-HY02-35_ = 699.952 ± 0.669 nm^2^, SASA_pTLR7-HY02-46_ = 700.973 ± 1.360 nm^2^) were all maintained at around 700 nm^2^ (Figure A1G–I and Table 4). This result indicates that ligand binding did not lead to substantial change in surface area at the binding interface nor did it change the burial or exposure of surface residues.

#### 2.7.5. Hydrogen Bonds

In order to investigate the hydrogen bonding properties of complexes, the number of hydrogen bonds between ligands and protein was calculated. As is known to all, in MD simulation, determining the number of hydrogen bonds can help understand the degree of interactions between molecules [51,52]. More hydrogen bonds between drug molecules and target usually mean that the interactions will be more firmly binding. As shown in Figure A1J,K, small molecules HY02-35 and HY02-46 formed no more than five and four hydrogen bonds with the receptor, respectively.

According to reports in the literature, amino acid residues in human TLR7, such as LYS432*, PHE408*, THR586, GLN354*, and VAL355, play important roles in receptor–ligand interactions. In our previous study, similar or consistent amino acid residues involved in interactions were also observed in mice and porcine. In addition, according to the results of MD simulation, it can be seen that THR587 and LEU351 act as hydrogen bond donors and acceptors, respectively, and dynamically contact with HY02-35. The low hydrogen bond occupancy indicates that it is mainly dynamic contact rather than anchored by a single persistent hydrogen bond. For HY02-46, LEU461 acts as the hydrogen bond donor, while VAL355, GLN354 and HIS559 are also key contributing factors (Table 5).

#### 2.7.6. DSSP Analysis

**Figure 9:**
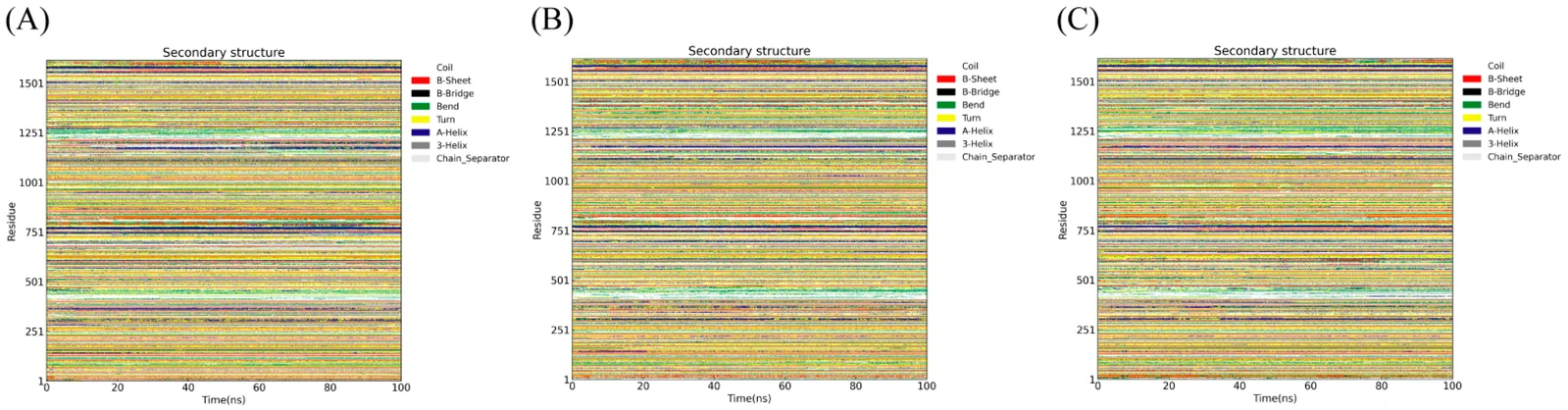
DSSP plot of apo-form (**A**); complexes of pTLR7 with HY02-35 (**B**) and HY02-46 (**C**), respectively.

Furthermore, calculation of the definition of the secondary structure of proteins (DSSPs) was done to assess the changes in secondary structure upon ligands’ binding [53,54]. After binding with two small molecules, the secondary structure in the region between approximately 400 and 500 of the target protein underwent corresponding changes, transitioning dynamically among β-bend, β-turn, and random coil conformations (Figure 9). This corresponds to the distribution of amino acids involved in the receptor–ligand interactions.

#### 2.7.7. Gibbs Energy Landscape

Gibbs energy landscape characterizes the free energy changes of the complex during the MD simulation phase. An energy trough represents the stable state of the system, which may correspond to the equilibrium configuration or the stable binding state between molecules [55,56]. Figure 8D–F display the Gibbs energy landscape. The blue area stands for the minimum energy value, indicating a more stable and thermodynamically favorable structure. On the contrary, the red region represents an instability of energy. Both complexes formed relatively single minimum energy clusters, suggesting good stability of receptor–ligand complexes.

#### 2.7.8. MM-PBSA

Binding free energies are depicted in Figure 8G,H. The average binding energies of complexes formed by HY02-35 and HY02-46 were −20.32 ± 3.54 and −17.13 ± 4.08 kcal/mol, respectively. Among them, ΔE_vdw_, ΔE_ele_, ΔG_GB_, and ΔG_Surf_ of HY02-35 were −30.27 ± 3.66, −14.24 ± 1.87, 28.43 ± 1.47, and −4.24 ± 0.44 kcal/mol, respectively. ΔE_vdw_, ΔE_ele_, ΔG_GB_, and ΔG_Surf_ of HY02-46 were −27.23 ± 4.66, −8.16 ± 3.50, 22.07 ± 3.78, and −3.80 ± 0.54 kcal/mol, respectively. Taking into account the above MD results, it was inferred that HY02-35 and HY02-46 remained at the binding site and formed stable complexes with the receptor protein during the 100 ns simulation.

### 2.8. Regulatory Effects on Cytokines’ Gene Expression

**Figure 10:**
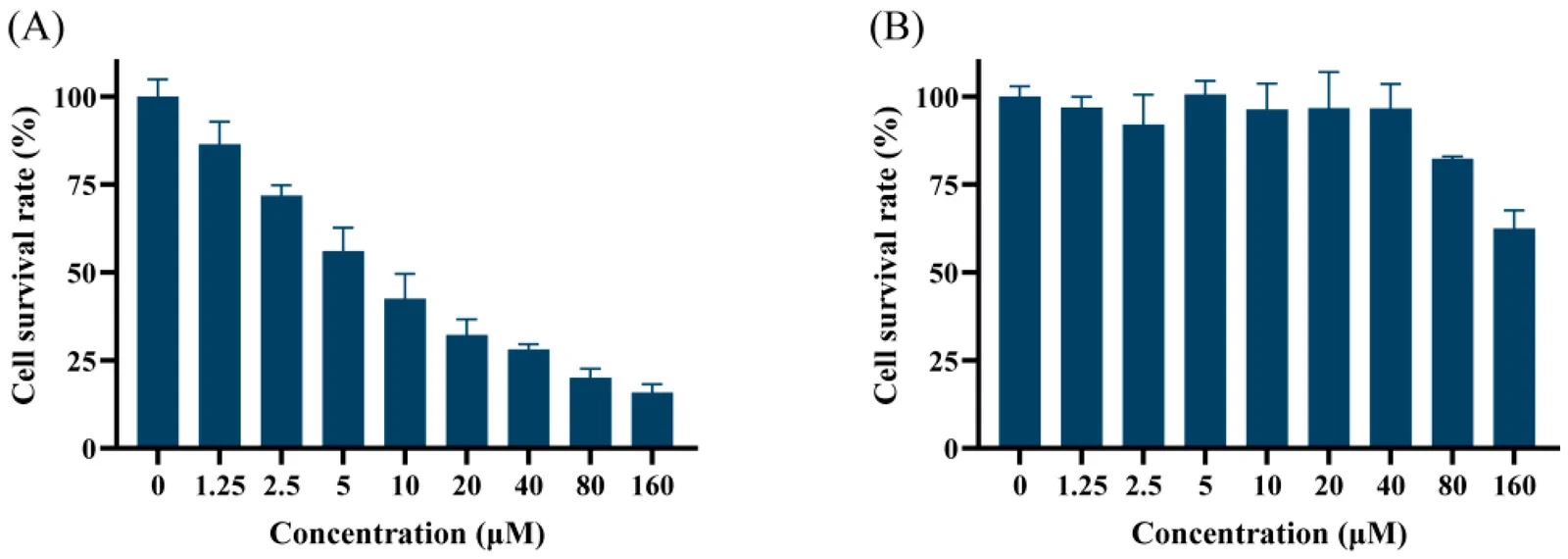
The cytotoxicity of hit compounds. (**A**,**B**) represent the cytotoxicity of HY02-35 and HY02-46 on THP-1 macrophages, respectively.

Prior to analyzing the gene expression of cytokines, the cytotoxicity of HY02-35 and HY02-46 on THP-1 macrophages was detected by the CCK-8 method. HY02-35 exhibits significant toxicity to THP-1 macrophages, with a cell survival rate of approximately 50% at the concentration of 5 μmol/L. In contrast, the toxicity of HY02-46 on THP-1 macrophages is relatively low (Figure 10).

**Figure 11:**
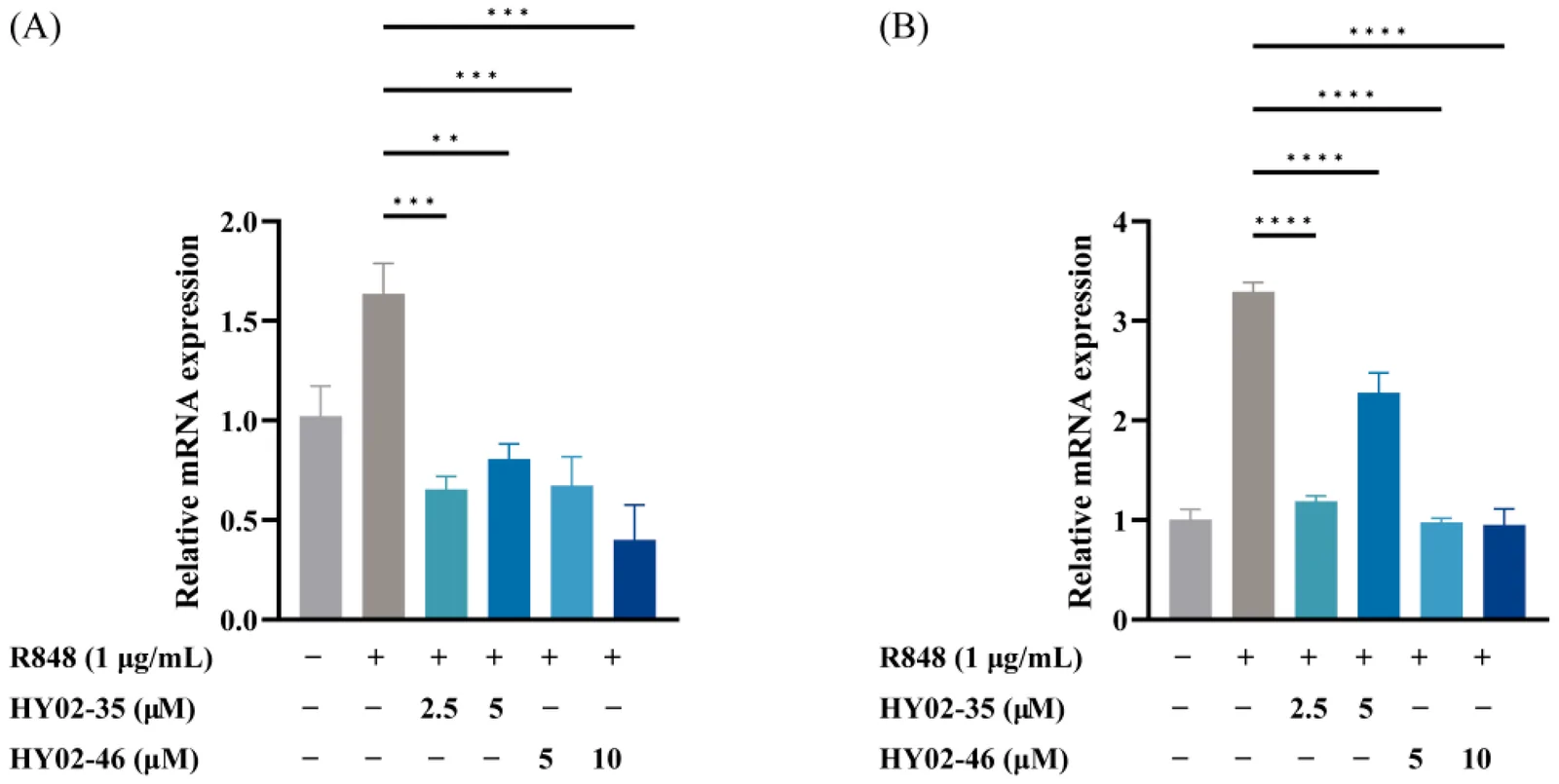
The regulatory effect on cytokines. (**A**,**B**) represent the effects of hit compounds on the gene expression of IFN-β and TNF-α in THP-1 macrophages, respectively. The data was normalized to GAPDH, and the results are presented as mean ± SD (*n* = 3). Statistical analysis was performed by one-way ANOVA with Dunnett’s post hoc test. ** represents *p* < 0.01, *** represents *p* < 0.001, and **** represents *p* < 0.0001.

Given the cytotoxicity and actual administration concentration, the regulatory effects of HY02-35 at concentrations of 2.5 μmol/L and 5 μmol/L, as well as HY02-46 at concentrations of 10 μmol/L and 20 μmol/L, on cytokine gene expression were investigated. TLR7 antagonists are well known in the treatment of various chronic inflammatory and autoimmune diseases. Previous studies have shown that TLR7 antagonists can inhibit the gene expression and production of inflammatory factors or cytokines, including IFN-α, IFN-β, TNF-α, IL-1β, IL-6, IL-17 and so on. As can be seen in Figure 11, R848 stimulated the gene expression levels of IFN-β and TNF-α in THP-1 macrophages. The mRNA levels of IFN-β and TNF-α can be reduced, to varying degrees, by HY02-35 and HY02-46, but not in a dose-dependent manner. The above results indicate that HY02-35 and HY02-46 have the potential to alleviate TLR7-induced inflammatory responses.

## 3. Discussion

The crystal structure of proteins provides crucial information for understanding protein function, elucidating protein–drug interactions, and for screening and designing of drugs [29]. Based on previous research, it is generally considered that TLR7 contains two different binding sites, which bind to small molecule ligands and ssRNA, respectively. TLR7 is induced to form dimers or conformational changes upon binding to ligands, thereby affecting downstream signal transduction [57,58]. In this study, the homology modeling method was applied, and the predicted structure of porcine TLR7 had good stereochemical properties and reasonable energy distribution, indicating that the model is reliable and can be used for subsequent analysis.

Molecular docking and ADMET prediction were used for preliminary screening. Understanding the physicochemical properties and pharmacokinetic characteristics of compounds can not only provide guidance for biological assays, administration routes and drug formulation design but also reduce the failure rate of drug development [35,37,38]. At this stage, small molecules with excellent physicochemical properties, pharmacokinetic characteristics and safety were screened out for subsequent experiments. It is worth mentioning that although the MD simulations provide insights into the dynamic behavior and stability of the receptor–ligand complexes, the absence of energy decomposition and residue contribution analyses limits the identification of specific residues and energetic contributions underlying ligand binding. Energy decomposition and residue contribution analysis can provide clear optimization directions and modifiable sites for drug design. They can also provide residue-level evidence for binding mechanism analysis, which can be used to explain differences in selectivity and binding modes between ligands.

Stable co-transfected [59,60] or transient co-transfection [26] with the target gene and reporter gene cell line, as well as peripheral blood mononuclear cells (PBMCs) [61], are common methods for evaluating the efficacy of TLR7 agonists/antagonists. Because of the lack of porcine TLR7 reporter cells, mouse TLR7 reporter cells were used to evaluate the regulatory effect of small molecules on TLR7. Two hit compounds, HY02-35 and HY02-46, exhibited a significant TLR7 inhibitory effect. But they also showed cytotoxicity at high concentrations, especially HY02-35.

Current research on TLR7 ligands mainly focuses on some classical structures, such as imidazoloquinoline, purine derivatives, etc. This study successfully screened two hits with structures different from the compounds reported in the literature, which has important research significance and application prospects. The novel structures provide a new scaffold for the development of TLR7 ligands, which can serve as a reference for drug design and help break through the limitations of current drug development and discover more promising drugs. Compared with reported antagonists, two hits exhibited moderate activity and cytotoxicity [62,63,64]. Strong activity and low toxicity are beneficial for enhancing the potential of compounds as candidate drugs. Structural modification could be considered in the future to improve the activity, as well as to reduce toxicity.

However, restricted by current practical conditions, there were some limitations in this study. Firstly, the success rate of drug screening is relatively low, and expanding the screening scope can improve the hit rate to a certain extent. Considering factors such as availability, cost, manpower, and time consumption, although 200,000 molecules were preliminary screened in the early stage, only fifty molecules were validated for TLR7 activity. The number of validated molecules was relatively limited. Secondly, TLR7 is mainly expressed in plasmacytoid dendritic cells (pDCs) and B cells, and PBMCs are usually applied to evaluate the immunomodulatory effect of TLR7 ligands. Here, considering the inconvenience of isolating and culturing primary cells, as well as the modest, but not outstanding, activity of hit compounds and their cytotoxicity, PBMCs were not used for validation at present. Further, in computational and experimental studies, mouse- and human-derived cells were used for activity evaluation, but there is still a lack of functional validation on porcine cells or in vivo. And the selectivity of two hits towards other TLRs has not been explored. The moderate activity and their observable cytotoxicity need to be addressed in subsequent studies. In addition, the lack of in vivo validation is one of the shortcomings of this study, as the in vivo efficacy and safety of hit compounds have not been evaluated. In future work, efforts will be made to develop more active ligands, and the regulatory function of TLR7 ligands on immune responses, and their ability to alleviate related diseases, will be analyzed. Extending these observations to the in vivo efficacy and safety evaluation of porcine and other livestock will be an essential next step.

## 4. Materials and Methods

### 4.1. Material and Reagents

Mouse TLR7 reporter cell and THP-1 cell were retrieved from Key Laboratory of Veterinary Pharmaceutical Development of Ministry of Agriculture and Rural Affairs, Lanzhou Institute of Husbandry and Pharmaceutical Sciences of Chinese Academy Agriculture Sciences. Fetal bovine serum (FBS, AUS-01S-02) was purchased from Cell-Box (Changsha, Hunan, China). Trypsin (25200072), RPMI 1640 medium (11875093) and EMEM medium were obtained from Gibco (Grand Island, NY, USA). Phorbol myristate acetate (PMA) (HY-18739) was bought from MedChemExpress LLC (Monmouth Junction, NJ, USA). Cell counting kit-8 (CCK-8) (C6005) was obtained from NCM Biotech (New cell & Molecular Biotech Co., Ltd., Suzhou, Jiangsu, China). One-Lumi firefly luciferase reporter gene assay kit (RG055M) was bought from Beyotime Biotech Inc. (Shanghai, China). TaKaRa MiniBEST Universal RNA extraction kit (9767), PrimeScript™ RT Master Mix (RR036A), and TB Green Premix Ex Taq™ II (RR820A) were purchased from TaKaRa Biotechnology (Dalian, China).

### 4.2. Sequence Retrieval and Alignment

Due to the lack of available crystal structures of porcine TLR7 in the PDB database, homology modeling was used to generate structural model based on proteins with similar sequences. The amino acid sequence of the target protein was retrieved and downloaded from the NCBI database (https://www.ncbi.nlm.nih.gov/) (accessed on 13 August 2024), with accession number ABC69202. A similarity search was performed using the Basic Local Alignment Search Tool (BLAST) in the NCBI database (https://blast.ncbi.nlm.nih.gov/Blast.cgi) (accessed on 13 August 2024) [30,65]. Sequence alignment between target protein and the modeling template was performed in Discovery Studio software [30]. Protein with high homology and known crystal structures in the PDB database was selected as modeling template [28].

### 4.3. Homology Modeling

Based on the selected modeling template, the three-dimensional (3D) structure of porcine TLR7 was predicted through the SWISS-MODEL online platform (https://swissmodel.expasy.org/) (accessed on 8 October 2024). The predicted model with higher GMQE (global model quality estimation) and QMEAN (qualitative model energy analysis) scores was selected and used for further in silico analyses [28,31]. PROCHECK module of Saves online platform (https://saves.mbi.ucla.edu/) (accessed on 8 October 2024), ProSA online platform (https://prosa.services.came.sbg.ac.at/prosa.php) (accessed on 8 October 2024) [66], and Verify Protein (Profiles-3D) module in Discovery Studio software (version 4.0) were used to evaluate the quality and reliability of the predicted homology model [31,67,68].

### 4.4. Molecular Docking and Virtual Screening

The Schrödinger suite was used to process receptor and ligands separately and for subsequent virtual screening [69]. Due to the lack of copyright, this section has been entrusted to MedChemExpress LLC, but the experimental design and result analysis are still carried out by us.

Protein Preparation Wizard module was applied to process missing atoms or structural elements and to add hydrogen to proteins and optimize energy. H-Bond position was optimized. Water molecules and heteroatoms were removed. The pH was set to 7.0–7.4. OPLS2005 force field was engaged, and the root mean square deviation (RMSD) value was 0.30  Å. The protein porcine TLR7 was defined as the receptor. Receptor Grid Generation module was used to create a grid file according to known agonists, with a box size of 20 Å × 20 Å × 20 Å [70].

Specs HTS Compounds Library (MedChemExpress, Monmouth Junction, NJ, USA), which contains 207,877 compounds, was selected as the ligand library. LigPrep Module module in Schrödinger Maestro 11.4 was employed to process small molecules in Specs HTS Compounds Library, and the 3D structure was output for virtual screening. Hydrogen was added, energy minimization was performed, force field was enabled, and the 3D structures were output for virtual screening.

Virtual Screening Workflow was adopted for virtual screening. Prepared molecules were imported, and Glide tool was used for molecular docking, where the receptor and ligands dock with each other through geometric and energy matching [69]. High-throughput virtual screening (HTVS) mode in the Glide tool was used to screen prepared compounds. The top 5% of small molecules ranked by scores were selected for second round of screening based on standard precision (SP) mode. Subsequently, the top 10% of compounds were picked out and subjected to a third round of filtering in extra precision (XP) mode. The ligand was set to generate up to 10 conformations in each docking run, while the remaining parameters remained default. The results were sorted according to the docking score.

### 4.5. ADMET and Drug-likeness Properties Prediction

Further screening of compounds was based on Lipinski’s rule of five and Veber rules. In Discovery Studio software, Prepare or Filter Ligands module was employed to evaluate the physicochemical properties of the screened compounds based on above rules, and structures with poor drug properties were excluded.

ADMETs are important pharmacokinetic parameters, and predicting ADMET properties is a crucial step in drug development [36]. Using the ADMET prediction module in Discovery Studio software [71], and/or SwissADME [40,72] (https://swissadme.ch/) (accessed on 17 January 2025) and ADMETlab 3.0 (https://admetlab3.scbdd.com/server/screening) (accessed on 17 January 2025) [73,74,75], molecules were evaluated and screened from several aspects, including physicochemical properties and pharmacokinetic characteristics, as well as safety. In DS software, the calculate molecular properties tool in the Small Molecules module was employed, where small molecules were inputted and ADMET descriptors such as aqueous solubility, blood–brain barrier (BBB) penetration, hepatotoxicity, and intestinal absorption were calculated. Several small molecules were submitted to the SwissADME and ADMETlab 3.0 platforms for a more comprehensive understanding of their properties.

### 4.6. Molecular Dynamics Simulation and Calculation of MM-PBSA

Molecular dynamic (MD) simulation is a molecular simulation method based on Newton’s laws of motion, which logically proves the conformation and molecular behavior at the atomic level and can infer various properties of the system [76]. GROMACS software (version 2022.6) was used for MD simulation. The stimulation protocols were moderately adjusted based on the literature.

Topology files of receptor and ligand were generated. The protein topology was pretreated using CHARMM36 force field. The ACPYPE tool was employed to calculate the atomic charges of ligands, and CHARMM General Force Field (CGen FF) was employed to generate topology files of small molecules [77]. TIP3P water model was employed for solvation, and a dodecahedral water box with a periodic boundary of 1.2 nm was established to avoid surface artifacts [78].

Long-range electrostatic interactions were computed using the PME (particle mesh Ewald) method, and neighbor searching was implemented using Verlet cutoff scheme [79]. Chloride and sodium ions with final concentration of 0.15 mol/L were utilized for system neutralization [80,81]. The linear constraint solver (LINCS) algorithm was applied to constrain all bonds. Energy minimization was conducted through steepest descent method in 50,000 steps, which aims to eliminate unreasonable conformations, unfavorable atomic contact and high-energy states in initial structure. After energy minimization, NVT and NPT equilibrium were executed to reach stability. The number of particles, volume, and temperature (310 K) were maintained constant, and 50,000 pre-equilibration steps with a time step of 2 fs were performed. Restraints on the atomic coordinates of protein and ligands were imposed by employing -DPOSRES in the parameter file. Then, the number of particles, pressure (1 bar), and temperature (310 K) were maintained constant, and 50,000 pre-equilibration steps with a time step of 2 fs were performed. In addition, 100 ns MD simulation was conducted with a time step of 2 fs [82].

Further, the binding energy was calculated based on molecular mechanics/Poisson–Boltzmann (generalized born) surface area (MM-PBSA) method. MM-PBSA method is one of the commonly used techniques for calculating the interaction energy between biomolecule complexes. Combined with MD simulation, MM-PBSA can explain significant structural changes and entropy binding energy contributions [83,84,85]. The gmx_MPBSA tool in GROMACS software was employed to calculate the binding free energy of the complex. The trajectory after the system reached equilibrium (60–100 ns) was used, with 1001 frames extracted at 40 ps intervals. Average binding free energy (ΔG_bind_) of complexes in solvent was calculated according to the following equation, with ΔG unit in Kcal/mol:

ΔG_bind_ = ΔE_MM_ + ΔG_GB_ + ΔG_Surf_ = ΔE_vdw_ + ΔG_ele_ + ΔG_GB_ + ΔG_Surf_ (2)

where ΔG_bind_ is average binding free energy of protein–ligand complexes; ΔE_MM_ is the molecular mechanics energy, including ΔE_vdw_ (van der Waals energy) and ΔE_ele_ (electrostatic energy); ΔG_GB_ and ΔG_Surf_ represent the polar electrostatic solvation energy and the nonpolar component, respectively [86,87].

### 4.7. CCK8 Method for Cell Viability Assay

Mouse TLR7 reporter cells were seeded into 96-well plates at a concentration of approximately 5 × 10^5^ cells/mL. When the cell fusion degree reached 50%, cells were treated with corresponding concentrations of test molecules and/or R848, respectively. After 24 h of treatment, CCK-8 reagent was added to each well, the material was incubated for 4 h, and the absorbance at 450 nm was measured. Each group had at least three repetitions. THP-1 cells were cultured in RPMI 1640 medium containing 10% FBS and 0.05 mmol/L β-mercaptoethanol and were induced with 50 ng/mL PMA before administration treatment.

### 4.8. Luciferase Assay

Mouse TLR7 reporter cells were seeded into 96-well plates at a concentration of approximately 5 × 10^5^ cells/mL. At the same time as above-mentioned experiment, cells were administered corresponding drugs by the same method, with R848 as the positive control. Following 24 h of incubation, according to the operating instruction of One-Lumi firefly luciferase assay kit, detection reagent was added, and luminescence was detected by Perkin Elmer ENSPIRE multifunctional plate reader. Results of treatment group were normalized against negative control. The calculation of IC_50_ was implemented using GraphPad Prism 9.0 software. Four-parameter nonlinear regression model was used for fitting the dose–response curve. The IC_50_ value and 95% CI of were calculated. Each group had at least three biological repetitions.

### 4.9. RNA Extraction and Quantitative Real-Time PCR

THP-1 cells were induced with 50 ng/mL PMA before administration of treatment. Afterwards, total RNA was extracted from three independent cell preparations using an RNA extraction kit and then was reverse transcribed into cDNA. The operating method was modified according to the instructions. Quantitative real-time PCR was conducted to analyze the relative gene expression levels under reaction conditions of 95 °C for 30 s, 95 °C for 5 s, and 60 °C for 34 s (40 cycles). Each sample was analyzed in triplicate as technical replicates. The primers were synthesized by Beijing Qingke Biotechnology Co., Ltd. (Beijing, China). (Table A3). The data was normalized to glyceraldehyde-3-phosphate dehydrogenase (GAPDH) and was analyzed in 2^−ΔΔCt^ method [88].

### 4.10. Statistic Analysis

The experiments were conducted in triplicate biological repeats. Each biological replicate includes three technical replicates, and the average of these technical replicates was used for statistic analysis. The results were represented as mean ± SD. Statistical analysis was conducted through one-way ANOVA multiple comparisons, followed by Dunnett’s post hoc test. * represents *p* < 0.05, ** represents *p* < 0.01, *** represents *p* < 0.001, and **** represents *p* < 0.0001.

## 5. Conclusions

In this study, two hit compounds with potential TLR7 inhibitory activity and novel parent nucleus structures were identified. Two small molecules, HY02-35 and HY02-46, exhibited significant inhibitory activity against mouse TLR7 but also exhibited a certain degree of cytotoxicity. Both of them participate in the formation of hydrogen bonds, electrostatic interactions, van der Waals forces, and hydrophobic interactions with key amino acid residues of porcine TLR7. The MD simulation results showed that the complexes formed between the two compounds and porcine TLR7 did not undergo significant conformational changes during the 100 ns simulation period, and the systems tended towards equilibrium and stability. According to the results of MM-PBSA, the average binding free energies of HY02-35 and HY02-46 with porcine TLR7 were −20.32 ± 3.54 and −17.13 ± 4.08 kcal/mol, respectively, implying that they have high binding affinity with porcine TLR7 and can bind to porcine TLR7 spontaneously. In addition, the two hit compounds can significantly suppress the R848-induced upregulation of cytokines at transcription levels in THP-1 cells.

Current research not only provides a starting point for the research of candidate TLR7 antagonists but also supplies an important reference for subsequent structure optimization and activity evaluation. Notably, the cell-based activity of HY02-35 and HY02-46 was assessed in mouse and human cell lines. The inhibitory potential of HY02-35 and HY02-46 against porcine TLR7 warrants further validation. The two hit compounds could currently be considered as molecules with in-depth research value; however, further research is required to determine their effectiveness, selectivity, safety, and pharmacokinetic characteristics in porcine cells or in vivo.

## Figures and Tables

**Table 1:** The biological activity and predicted properties of two hit compounds, including docking score, IC_50_, and predicted features of ADMETlab 3.0 and SwissADME.

Compound ID	Docking Score (kcal/mol)	IC_50_ (μmol/L)	GI Absorption	P-gp Substrate	Log P	TPSA (Å^2^)
HY02-35	−10.53	14.73 (95% CI: 10.25–38.23)	High	No	1.71	111.07
HY02-46	−10.28	26.61 (95% CI: 21.92–36.00)	High	No	3.49	87.82

**Table 2:** Average and standard deviation (SD) of RMSD between 60 ns and 100 ns.

Complex	Replicate 1 (nm)	Replicate 2 (nm)	Replicate 3 (nm)	Mean ± SD (nm)
pTLR7-apo	0.325 ± 0.024	0.328 ± 0.017	0.380 ± 0.018	0.344 ± 0.004
pTLR7-HY02-35	0.386 ± 0.019	0.380 ± 0.017	0.388 ± 0.014	0.385 ± 0.003
pTLR7-HY02-46	0.363 ± 0.026	0.466 ± 0.023	0.352 ± 0.014	0.394 ± 0.006

**Table 3:** Mean ± SD of Rg between 60 ns and 100 ns.

Complex	Replicate 1 (nm)	Replicate 2 (nm)	Replicate 3 (nm)	Mean ± SD (nm)
pTLR7-apo	3.886 ± 0.013	3.881 ± 0.012	3.888 ± 0.013	3.885 ± 0.001
pTLR7-HY02-35	3.906 ± 0.011	3.906 ± 0.011	3.871 ± 0.011	3.894 ± 0.000
pTLR7-HY02-46	3.888 ± 0.012	3.925 ± 0.013	3.877 ± 0.012	3.897 ± 0.001

**Table 4:** Mean ± SD of SASA between 60 ns and 100 ns.

Complex	Replicate 1 (nm^2^)	Replicate 2 (nm^2^)	Replicate 3 (nm^2^)	Mean ± SD (nm^2^)
pTLR7-apo	694.937 ± 5.758	704.067 ± 6.605	700.034 ± 7.411	699.679 ± 0.841
pTLR7-HY02-35	706.736 ± 6.815	705.147 ± 5.755	687.972 ± 6.922	699.952 ± 0.669
pTLR7-HY02-46	693.845 ± 7.805	712.363 ± 5.383	696.712 ± 5.523	700.973 ± 1.360

**Table 5:** Hydrogen bond occupancy between protein and ligands over the 100 ns MD simulation.

Complex	Donor	Acceptor	Occupancy (%)
pTLR7-HY02-35	THR587@N (Main)	LEU351@O2 (Side)	9.91%
pTLR7-HY02-35	THR587@N (Main)	LEU351@O1 (Side)	8.48%
pTLR7-HY02-35	THR587@N (Main)	LEU351@N4 (Side)	7.78%
pTLR7-HY02-35	ARG262@NH_2_ (Side)	LEU351@N4 (Side)	6.77%
pTLR7-HY02-35	THR587@N (Main)	LEU351@N1 (Side)	4.17%
pTLR7-HY02-46	LEU461@N2 (Side)	VAL355@O (Main)	10.31%
pTLR7-HY02-46	LEU461@N4 (Side)	GLN354@O (Main)	9.82%
pTLR7-HY02-46	LEU461@N2 (Side)	GLN354@OE1 (Side)	7.33%
pTLR7-HY02-46	LEU461@N4 (Side)	HIS559@ND1 (Side)	7.11%
pTLR7-HY02-46	ARG410@NH_2_ (Side)	LEU461@N3 (Side)	4.38%

## Data Availability

The data presented in this study are available from the corresponding author upon reasonable request.

## Abbreviations

The following abbreviations are used in this manuscript:

TLR	Toll-like receptor
PRR	Pattern recognition receptor
PAMP	Pathogen-associated molecular pattern
DAMP	Damage-associated molecular pattern
TIR	Toll/interleukin-1 receptor
MAPK	Mitogen-activated protein kinase
PI3K/AKT	Phosphoinositol 3-kinase/serine/threonine protein kinase
R837	Imiquimod
ADMET	Absorption, distribution, biotransformation, excretion, and toxicity
MD simulation	Molecular dynamics simulation
PME	Particle mesh Ewald
FBS	Fetal bovine serum
CCK-8	Cell counting kit-8
BLAST	Basic local alignment search tool
GMQE	Global model quality estimation
QMEAN	Qualitative model energy analysis
RMSD	Root mean square deviation
HIA	Human intestinal absorption
BBB	Blood–brain barrier
MM-PBSA	Molecular mechanics/Poisson–Boltzmann surface area
R848	Resiquimod
MW	Molecular weight
HBDs	Hydrogen bond donors
HBAs	Hydrogen bond acceptors
LogP	Partition coefficient
TPSA	Topological polar surface area
LD_50_	Median lethal dose
IC_50_	Half-maximal inhibitory concentration
RMSF	Root mean square fluctuation
Rg	Radius of gyration
SASA	Solvent accessible surface area
NHB	Hydrogen bond number
PBMCs	Peripheral blood mononuclear cells
pDCs	Plasmacytoid dendritic cells

## Appendix A

**Table A1:** Docking score and physicochemical properties of 50 screened small molecules.

Compound ID	Docking Score (kcal/mol)	Molecular Weight	H-Bond Donors	H-Bond Acceptors	LogP	Rotatable Bonds	TPSA
HY02-31	−9.98	257.72	1	3	2.59	3	48.71
HY02-32	−12.16	247.29	2	4	1.04	4	66.4
HY02-33	−9.43	321.39	0	5	3.31	6	84.08
HY02-34	−9.44	284.27	1	2	2.8	4	92.8
HY02-35	−9.41	292.25	1	4	2.14	2	108.39
HY02-36	−9.86	298.3	3	4	0.5	2	117.18
HY02-37	−9.59	376.25	0	4	2.23	4	45.67
HY02-38	−9.50	380.44	3	7	1.9	7	146.25
HY02-39	−10.70	252.65	1	4	1.17	2	63.6
HY02-40	−11.02	332.39	0	5	3.17	11	69.67
HY02-41	−10.47	397.42	1	4	5.92	6	76.49
HY02-42	−9.93	277.27	2	4	2.12	4	84.86
HY02-43	−9.82	281.31	2	5	0.94	3	100.61
HY02-44	−10.86	324.38	2	4	2.59	3	96.95
HY02-45	−10.84	312.34	2	4	2.71	2	87.72
HY02-46	−10.28	294.35	2	4	2.51	2	87.72
HY02-47	−10.28	295.34	2	5	1.32	2	100.61
HY02-48	−12.91	285.29	2	4	1.77	3	79.54
HY02-49	−13.52	291.69	2	4	1.32	3	79.54
HY02-50	−11.18	391.37	1	4	2.27	5	89.21
HY02-51	−11.41	407.42	1	4	1.85	7	89.21
HY02-52	−10.87	375.42	1	4	3.3	5	70.75
HY02-53	−10.27	258.27	0	3	2.89	5	56.51
HY02-54	−9.59	337.33	1	5	1.88	8	85.61
HY02-55	−10.86	419.21	0	4	4.13	8	82.81
HY02-56	−9.81	380.19	2	6	0.46	4	92.7
HY02-57	−9.44	325.32	3	6	−0.28	4	140.04
HY02-58	−10.51	333.39	3	5	2.69	4	103.51
HY02-59	−9.95	427.47	2	6	3.1	8	156.62
HY02-60	−10.37	345.4	2	5	2.77	4	100.61
HY02-61	−9.67	322.36	1	3	3.55	4	56.51
HY02-62	−10.68	469.45	1	4	2.83	7	133.46
HY02-63	−9.74	309.2	1	3	4.64	5	41.99
HY02-64	−9.62	242.27	2	4	1.93	4	62.22
HY02-65	−10.53	299.32	2	3	3.17	4	67.79
HY02-66	−11.25	301.36	2	4	2.41	4	94.64
HY02-67	−10.44	369.64	1	4	2.98	7	51.22
HY02-68	−9.90	301.3	1	4	99.99	1	75.01
HY02-69	−10.56	322.31	2	3	4.14	5	83.83
HY02-70	−10.39	236.2	1	4	0.73	2	63.6
HY02-71	−9.48	290.34	2	5	2.64	4	83.65
HY02-72	−10.22	396.42	2	7	4.03	5	112.16
HY02-73	−9.98	382.39	2	7	3.54	4	112.16
HY02-74	−9.83	357.38	1	4	1.71	5	101.37
HY02-75	−9.98	377.39	2	6	3.21	5	95.52
HY02-76	−10.33	344.79	1	5	3.82	4	60.85
HY02-77	−9.61	316.37	1	5	3	4	89.09
HY02-78	−10.42	324.37	1	5	3.73	4	60.85
HY02-79	−9.76	328.34	1	5	3.38	4	60.85
HY02-80	−9.97	270.26	2	5	2.16	1	81.57

**Table A2:** ADMET properties and predicted rat oral LD_50_ of 50 screened small molecules.

Compound ID	ADMET Solubility Level ^a^	ADMET BBB Level ^b^	ADMET EXT Hepatotoxic# Prediction	ADMET Absorption Level ^c^	ADMET EXT PPB# Prediction	Rat Oral LD_50_ (g/kg)
HY02-01	3	1	TRUE	0	TRUE	1.43
HY02-02	3	3	FALSE	0	TRUE	1.09
HY02-03	3	3	TRUE	0	TRUE	2.06
HY02-04	3	3	TRUE	0	TRUE	0.53
HY02-05	3	3	TRUE	0	FALSE	1.25
HY02-06	3	4	TRUE	0	FALSE	0.96
HY02-07	3	2	TRUE	0	TRUE	1.09
HY02-08	4	4	TRUE	0	TRUE	5.63
HY02-09	3	3	TRUE	0	TRUE	0.82
HY02-10	3	2	FALSE	0	TRUE	1.33
HY02-11	2	1	TRUE	0	TRUE	4.39
HY02-12	4	3	TRUE	0	TRUE	1.18
HY02-13	3	3	TRUE	0	FALSE	1.05
HY02-14	2	3	TRUE	0	TRUE	1.37
HY02-15	2	2	TRUE	0	TRUE	0.59
HY02-16	2	2	TRUE	0	TRUE	0.75
HY02-17	2	3	TRUE	0	FALSE	0.65
HY02-18	3	3	TRUE	0	TRUE	5.53
HY02-19	3	3	TRUE	0	TRUE	0.90
HY02-20	3	3	FALSE	0	TRUE	3.60
HY02-21	3	3	FALSE	0	TRUE	11.69
HY02-22	2	2	FALSE	0	TRUE	5.41
HY02-23	2	2	TRUE	0	TRUE	1.08
HY02-24	3	3	TRUE	0	FALSE	1.45
HY02-25	2	4	TRUE	0	TRUE	0.54
HY02-26	3	3	TRUE	0	FALSE	2.89
HY02-27	3	4	TRUE	1	FALSE	0.69
HY02-28	2	4	TRUE	0	FALSE	1.82
HY02-29	3	3	TRUE	0	TRUE	0.71
HY02-30	2	4	TRUE	0	FALSE	0.63
HY02-31	3	2	TRUE	0	TRUE	0.70
HY02-32	2	2	FALSE	0	TRUE	2.26
HY02-33	3	1	TRUE	0	TRUE	6.57
HY02-34	4	3	TRUE	0	TRUE	0.72
HY02-35	3	3	TRUE	0	TRUE	0.74
HY02-36	3	3	TRUE	0	TRUE	1.57
HY02-37	3	2	TRUE	0	TRUE	22.65
HY02-38	3	3	TRUE	0	FALSE	0.60
HY02-39	2	2	TRUE	0	TRUE	2.21
HY02-40	3	3	TRUE	0	TRUE	1.20
HY02-41	3	3	TRUE	0	TRUE	5.08
HY02-42	3	3	TRUE	0	TRUE	3.72
HY02-43	3	3	TRUE	0	TRUE	1.74
HY02-44	3	3	TRUE	0	TRUE	4.39
HY02-45	2	3	FALSE	0	FALSE	4.74
HY02-46	3	3	TRUE	0	TRUE	2.79
HY02-47	3	3	TRUE	0	TRUE	7.02
HY02-48	3	3	FALSE	0	TRUE	10.51
HY02-49	3	3	TRUE	0	TRUE	1.42
HY02-50	3	3	TRUE	0	TRUE	0.73

^a^: ADMET solubility level reflects the water solubility of compounds, ranging from 0 to 6. The larger the value, the higher the solubility. But excessive solubility is disadvantageous. ^b^: BBB, blood–brain barrier. ADMET BBB level ranges from 0 to 5. The smaller the value, the greater the ability to penetrate the blood–brain barrier. ^c^: The higher the value of ADMET absorption level, the more difficult it is to absorb. A value of 0 represents good absorption.

**Table A3:** Primer sequence for quantitative real-time PCR.

Gene		Primer Sequence (5′–3′)
IFN-β	Forward	AGGACAGGATGAACTTTGAC
	Reverse	TGATAGACATTAGCCAGGAG
TNF-α	Forward	CCCAGGGACCTCTCTCTAATC
	Reverse	ATGGGCTACAGGCTTGTCACT
GAPDH	Forward	GGACAGGATATGGAGCAACAAGTGG
	Reverse	TCATCTTTCAACACGCAGGACAGG
